# Early peak temperature and mortality in critically ill patients with or without infection

**DOI:** 10.1186/cc10393

**Published:** 2011-10-27

**Authors:** M Saxena, P Young, R Beasley, M Bailey, R Bellomo, D Pilcher, S Finfer, D Harrison, J Myburgh, K Rowan

**Affiliations:** 1 George Institute for Global Health, Sydney, NSW, Australia; 2 St George Clinical School, University of New South Wales, Sydney, NSW, Australia; 3 Intensive Care Unit, Wellington Regional Hospital, Capital and Coast District Health Board, Wellington, New Zealand; 4 Medical Research Institute of New Zealand, Wellington, New Zealand; 5 Australian and New Zealand Intensive Care Research Centre, School of Public Health and Preventive Medicine, Monash University, Melbourne, VIC, Australia; 6 Australian and New Zealand Intensive Care Society Centre for Outcome and Resource Evaluation, Melbourne, VIC, Australia; 7 Sydney Medical School, University of Sydney, NSW, Australia; 8 Intensive Care National Audit and Research Centre, London, UK

## Introduction

The febrile response in the context of infection may be linked to a protective host response through enhanced immune function at elevated body temperatures [1-9]. Alternatively the use of antipyretics may reduce metabolic expense, patient discomfort, or protect against neurological injury.

## Objective

To determine whether fever is associated with reduced risk of death in patients admitted to an ICU with infection compared with other patients.

## Methods

A retrospective cohort study using a database of Australian and New Zealand (ANZ) ICU admissions as a development cohort and a database of UK ICU admissions as a validation cohort. The sample included 129 ICUs in ANZ and 201 ICUs in the UK. The ANZ development cohort consisted of 269,078 patients and the UK validation cohort consisted of 366,973 patients. All patients were admitted to an ICU between 2005 and 2009. A total of 29,083/269,078 (10.8%) ANZ patients and 103,191/366,973 (28.1%) UK patients were categorised as having an infection at the time of ICU admission. The main outcome measures were the association between peak temperature in the first 24 hours after ICU admission and in-hospital mortality in patients admitted with or without infection.

## Results

**Figure 1:**
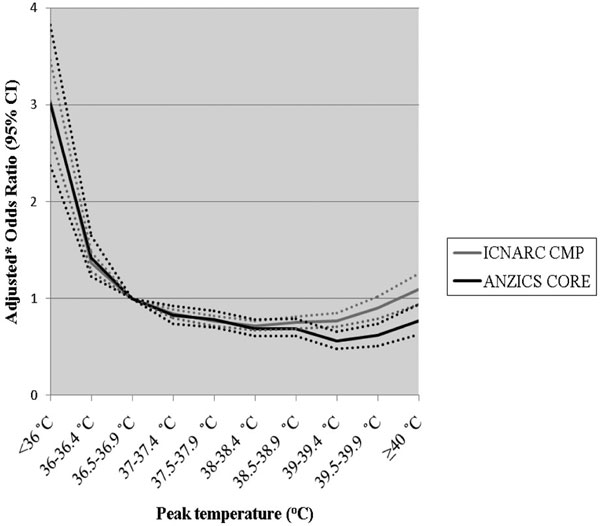
Adjusted* odds ratios for in-hospital mortality versus peak temperature in the first 24 hours in the ICU for patients in the infection group. *Odds ratios adjusted for illness severity using the ICNARC (2009) model predicted log odds of acute hospital mortality with the temperature component removed for the UK data and the APACHE III predicted log odds risk of death with the temperature component removed for the ANZ data.

**Figure 2:**
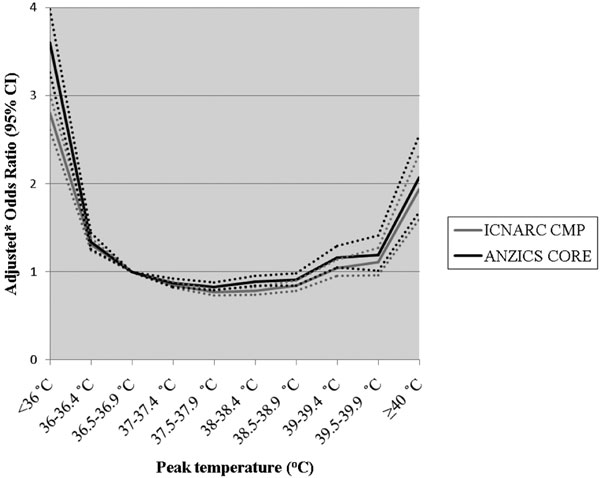
Adjusted* odds ratios for in-hospital mortality versus peak temperature in the first 24 hours in the ICU for patients in the non-infection group. *Odds ratios adjusted for illness severity using the ICNARC (2009) model predicted log odds of acute hospital mortality with the temperature component removed for the UK data and the APACHE III predicted log odds risk of death with the temperature component removed for the ANZ data.

In the ANZ cohort, adjusted in-hospital mortality risk progressively decreased with increasing peak temperature in patients with infection. Relative to 36.5 to 36.9°C, the lowest risk was at 39 to 39.4°C (adjusted OR = 0.56; 95% CI = 0.48 to 0.66). In patients without infection, the adjusted mortality risk progressively increased above 39.0°C (adjusted OR = 2.07 at ≥40.0°C; 95% CI = 1.68 to 2.55). In the UK cohort, findings were similar with adjusted odds ratios at corresponding temperatures of 0.77 (95% CI = 0.71 to 0.85) and 1.94 (95% CI = 1.60 to 2.34) for the infection and non-infection groups, respectively. See Figures 1 and 2.

## Conclusion

Peak temperature in the first 24 hours in the ICU is associated with decreased in-hospital mortality in critically ill patients with an infection; randomised trials are needed to compare the effect on mortality of controlling fever against a permissive approach to fever management in such patients.
